# In Memoriam: David Mark Baguley

**DOI:** 10.3390/audiolres12060057

**Published:** 2022-10-24

**Authors:** Don McFerran, Laurence McKenna

**Affiliations:** 1 British Tinnitus Association, Woodseats Close, Sheffield S8 0TB, UK; 2 Department of Clinical Psychology, Royal National ENT and Eastman Dental Hospitals, University College Hospital, 47–49 Huntley St, London WC1E 6DG, UK

Reverend Professor David (Dave) Mark Baguley, audiologist, hearing scientist, tinnitus clinician, educator, and Church of England priest, died suddenly and unexpectedly in Nottingham, UK on 11 June 2022, at the age of 61 (Figure 1). Dave was preceded in death by his mother Sheila. He is survived by his wife Bridget; their children Sam, Naomi and Luke; his father, Philip and his brothers Peter and Richard.

He was born on the 18 March 1961 in Manchester, UK. The family relocated to Ipswich, Suffolk but Dave never lost his Mancunian roots, and remained a fervent lifelong supporter both of Manchester City soccer team and of Manchester Indie Music. After attending Northgate Grammar School for Boys in Ipswich (now Northgate High School) he returned to study at Manchester University where he was awarded a BSc (Hons) in Psychology in 1983, and subsequently an MSc in Clinical Audiology in 1985.

After university, his first job was as Scientific Officer at the Medical Research Council (MRC) Institute of Hearing Research in Cardiff, Wales. After eight months in this post, he moved to Addenbrookes Hospital in Cambridge UK, working for the National Health Service (NHS) as an Audiological Scientist. Four years later he became Head of Audiology and was later awarded Consultant status. He remained at Addenbrookes Hospital for over 30 years and the Audiology Department grew under his stewardship to achieve international renown. Latterly, he added Head of Hearing Implants to his job description and became Clinical Lead for the local neonatal hearing screening service. As part of the process of developing the Audiology Department, he instigated Cambridge’s first dedicated Tinnitus Clinic in 1987.

To support his growing managerial responsibilities, he studied for an MBA which was awarded with Distinction by the Open University in 1994. His academic career was also blossoming and by this stage his medical writing was in full flow. He was contributing to the peer-reviewed medical press at a prodigious rate and ultimately wrote more than 220 scientific articles. The main thrust of his initial research and writing concerned vestibular schwannomas but by the late 1990s his research direction had shifted, and the publication emphasis became tinnitus and hyperacusis. Although he wrote many erudite papers, he seemed most fond of some of his quirkier publications. Finding a niche that no-one else had considered gave him great pleasure and resulted in one paper about positive experiences of tinnitus, another about the international vocabulary of tinnitus and a book chapter on tinnitus and hyperacusis in literature, film, and music. This offbeat approach spilled into his presentations which were often scattered with tinnitus references from literature and the arts: even Tintin featuring in one of his talks! While continuing to work full-time, he undertook a PhD on the physiological mechanisms of tinnitus in patients with vestibular schwannoma. This degree was awarded by Cambridge University in 2005.

Throughout his tenure at Addenbrookes, Dave developed what was to become a lifelong passion for teaching, and this extended not only to audiology staff but also to both the homegrown medical trainees passing through the ENT Department and the international research fellows who were attached to the hospital’s skull base team. This ability to cross boundaries into other clinical and research disciplines was one of Dave’s strongest points: he was equally comfortable talking to a young audiologist, a senior ENT surgeon, a representative of big pharma, a psychologist or the head of a large university department. This skill, combined with an encyclopaedic knowledge of tinnitus and hyperacusis research, ensured that he became the go-to person for people wanting advice on new research topics or novel tinnitus treatments. It also ensured that he became an unusual entity: an audiologist who was comfortable speaking at big ENT events. He presented several times at the Otology section of the Royal Society of Medicine and the UK’s leading ENT conference, the British Academic Conference in Otolaryngology (BACO).

The ability to move seamlessly between clinical, research and industry settings made his opinion almost indispensable in matters of translational research: nearly all the recent trials of potential tinnitus drugs or therapeutic devices sought Dave’s views prior to commencement. He was passionate in his view that tinnitus should be approached as a team effort and that only by adopting a multidisciplinary approach could we hope to move forward.

Dave cared deeply about his work, and this is one of the factors that helped to make him such an extraordinary teacher. On hearing of Dave’s death one colleague said: “I can remember so clearly everything he taught me. It was impossible to attend a lecture he gave without coming away with your mind changed or challenged about something in a way that usually led to a more compassionate outlook or understanding. And somehow, he did it without making you wrong for having thought about it differently.” Dave taught on many courses, including the long-running European Tinnitus Course. He was always happy to have detailed and careful conversations about the content of his lectures, over coffee, lunch, even breakfast the following morning, or indeed, later via email. He was available to all. In the words of another colleague: “In the big scheme of things, no one would ever know me really, I’m just an everyday audiologist yet Dave always included me and reached out to share information or respond to questions as if I were important. He made me feel important.” His care was also very evident in his clinical work. At times this made him irascible if he felt others were not putting in the same effort, but these occasional outbursts were usually short lived, and he was adept at defusing such situations with a sprinkling of wit and charm.

In 2007, Dave took a brief sabbatical from Cambridge and for four months undertook the role of Raine/Phonak Visiting Professor at the University of Western Australia, Perth, Australia. A year after his return he was offered a UK University Chair and became Visiting Professor at Anglia Ruskin University in Cambridge and Chelmsford in 2009.

In addition to journal articles, Dave wrote countless book chapters and was editor for books on tinnitus, hearing loss and hyperacusis. He co-authored two tinnitus books: one textbook for professionals, Tinnitus, a multidisciplinary approach; one self-help book for people with tinnitus and hyperacusis, Living with tinnitus and hyperacusis. Both books were well received and are in their second editions.

Dave contributed to many organisations and committees at local, national, and international level. He was a regular speaker at the local tinnitus support group in Cambridge. He was Chair of the British Society of Audiology, 2009–2011, and was editor of their periodical, the British Journal of Audiology (now the International Journal of Audiology) from 1995–2000. His vision helped to create the British Academy of Audiology. Dave joined the Editorial Board of the journal ENT News in 2008 and was instrumental in expanding the remit of the journal to include audiology, resulting in a name change to ENT & Audiology News. He was a member and subsequently Chair of the Professional Adviser’s Committee of the British Tinnitus Association and served as its President from 2015 to 2019. He sat on a Department of Health committee developing tinnitus commissioning guidelines. Dave was involved in the formation of the international committee of the American Academy of Audiology and served as co-chair for three years.

Dave was the recipient of numerous prizes and awards, including the Marie and Jack Shapiro Research Prize of the British Tinnitus Association on no less than five occasions, the TS Littler Research Prize from the British Society of Audiology (1994), the International Award of the American Academy of Audiology (2006), the Golden Lobe Award from the Association of Independent Hearing Healthcare Practitioners (2016), and the Norman Gamble Research Prize from the Royal Society of Medicine (2018).

In 2016 Dave decided on a career change and relocated to the University of Nottingham, taking up the position of Professor of Hearing Sciences within the School of Medicine’s Division of Clinical Neuroscience. He was Deputy Lead of the Hearing Theme in the Nottingham National Institute for Health and Care Research (NIHR) Biomedical Research Centre. Projects that he was involved with included investigation of hearing loss and tinnitus following platinum-based chemotherapy, development of a hearing bioresource, and a clinical trial on new adult hearing aid users, funded by the Health Technology Assessment Programme.

From his 20s onwards Dave developed a deep Christian faith and in characteristic fashion he chose to take a very active role: in 2011 he received a Diploma in Pastoral Theology from Anglia Ruskin University and was ordained Deacon in the Church of England. In 2012 he was ordained at Ely Cathedral as Priest in the Church of England. When the family relocated to Nottingham, Dave’s wife Bridget took on the Ministry of St Martin’s Church, Sherwood and Dave became Associate Minister.

Music featured highly in Dave’s life. He had eclectic tastes that encompassed everything from Van Morrison to Lee Scratch Perry to The Broken Family Band. However, undoubtedly his greatest admiration was for the Manchester band Joy Division and its rebirth as New Order. He was a keen attender of live music events, particularly the annual Cherry Hinton Folk Festival. Dave was an enthusiastic musician himself, playing rhythm guitar and it was a great source of pride to him that his children had embraced his joy of music.

One might be forgiven for thinking that there would be little room for anything else in Dave’s life, but he had many other interests: voracious reader, talented cook, hill walker, family man and above all, he loved to sit and chat, preferably over a pint or two of real ale.

## Figures and Tables

**Figure 1:**
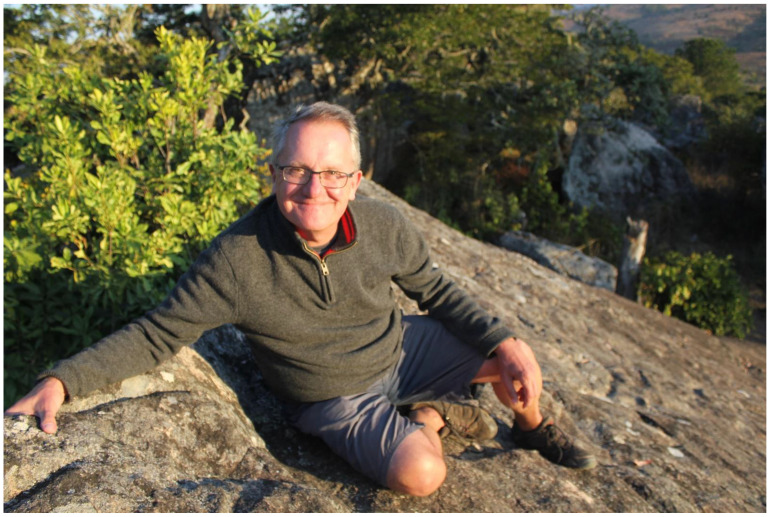
Photo of Professor David (Dave) Mark Baguley.

## Data Availability

Not applicable.

